# Using a theory of change in monitoring, evaluating and steering scale-up of a district-level health management strengthening intervention in Ghana, Malawi, and Uganda – lessons from the PERFORM2Scale consortium

**DOI:** 10.1186/s12913-022-08354-y

**Published:** 2022-08-05

**Authors:** Maryse Kok, Susan Bulthuis, Marjolein Dieleman, Olivier Onvlee, Rebecca Murphy, Patricia Akweongo, Justine Namakula, Hastings Banda, Kaspar Wyss, Joanna Raven, Tim Martineau

**Affiliations:** 1 KIT Royal Tropical Institute, Amsterdam, the Netherlands; 2 Athena Institute, VU University, Amsterdam, the Netherlands; 3 Department of Psychology, Maynooth University, Kildare, Ireland; 4 Department of Health Policy, Planning & Management, School of Public Health, College of Health Sciences, University of Ghana, Legon, Accra, Ghana; 5 Makerere University School of Public Health, Kampala, Uganda; 6 Research for Equity and Community Health (REACH) Trust, Lilongwe, Malawi; 7 Swiss Centre for International Health, Swiss Tropical and Public Health Institute, Allschwil, Switzerland; 8 University of Basel, Basel, Switzerland; 9 Department of International Public Health, Liverpool School of Tropical Medicine, Liverpool, UK

**Keywords:** Theory of change, Scale-up, Health management, Health system strengthening, Ghana, Malawi, Uganda

## Abstract

**Background:**

Since 2017, PERFORM2Scale, a research consortium with partners from seven countries in Africa and Europe, has steered the implementation and scale-up of a district-level health management strengthening intervention in Ghana, Malawi and Uganda. This article presents PERFORM2Scale’s theory of change (ToC) and reflections upon and adaptations of the ToC over time. The article aims to contribute to understanding the benefits and challenges of using a ToC-based approach for monitoring and evaluating the scale-up of health system strengthening interventions, because there is limited documentation of this in the literature.

**Methods:**

The consortium held annual ToC reflections that entailed multiple participatory methods, including individual scoring exercises, country and consortium-wide group discussions and visualizations. The reflections were captured in detailed annual reports, on which this article is based.

**Results:**

The PERFORM2Scale ToC describes how the management strengthening intervention, which targets district health management teams, was expected to improve health workforce performance and service delivery at scale, and which assumptions were instrumental to track over time. The annual ToC reflections proved valuable in gaining a nuanced understanding of how change did (and did not) happen. This helped in strategizing on actions to further steer the scale-up the intervention. It also led to adaptations of the ToC over time. Based on the annual reflections, these actions and adaptations related to: assessing the scalability of the intervention, documentation and dissemination of evidence about the effects of the intervention, understanding power relationships between key stakeholders, the importance of developing and monitoring a scale-up strategy and identification of opportunities to integrate (parts of) the intervention into existing structures and strategies.

**Conclusions:**

PERFORM2Scale’s experience provides lessons for using ToCs to monitor and evaluate the scale-up of health system strengthening interventions. ToCs can help in establishing a common vision on intervention scale-up. ToC-based approaches should include a variety of stakeholders and require their continued commitment to reflection and learning on intervention implementation and scale-up. ToC-based approaches can help in adapting interventions as well as scale-up processes to be in tune with contextual changes and stakeholders involved, to potentially increase chances for successful scale-up.

## Background

Human resources for health are essential in attaining universal health coverage. The World Health Organization, in its Global Strategy on Human Resources for Health, has put emphasis on improving health workforce performance, besides increasing the number of health workers [1]. While the size, composition and quality of the health workforce in the public health system are the responsibility of the national level, performance management of primary level staff is often a district-level responsibility [2]. Decentralization has led, to different extents, to increased decision space of district health management teams (DHMTs) in low- and middle-income countries, providing potential for improved human resource management [2–4]. However, not all district-level health managers have undergone management training [5, 6]. Therefore, strengthening of district-level health management is needed [7].

From 2011 to 2015, a district-level health management strengthening intervention (MSI) was piloted in nine districts in three countries in Sub-Saharan Africa. The intervention included a participatory action research approach, in which DHMTs conducted a plan-act-observe-reflect cycle related to a prioritized health workforce or service delivery problem. The MSI had positive effects on district health management and workforce performance [7]. Since 2017, the PERFORM2Scale consortium^1^ has been implementing and studying the scale-up of this MSI in Ghana, Malawi, and Uganda.

We define scale up as “deliberate efforts to increase the impact of successfully tested pilot, demonstration or experimental projects to benefit more people and to foster policy and programme development on a lasting basis” [8]. Several scale-up frameworks outline steps and strategies for successful scale-up [9–11]. PERFORM2Scale has been using an adapted version of the ExpandNet framework for the scale-up of the MSI, in which characteristics of the intervention, the stakeholders who use and steer it, the broader environment and the scale-up strategy interact and influence scale-up [11]. Over recent years, several studies have assessed factors that influence scale-up, such as engaging key stakeholders and having a well-defined scale-up strategy [12, 13]. Despite this, there is limited guidance on how to scale up [14] and on how scale-up can be monitored and evaluated to extract learnings for scale-up of future interventions.

A theory of change (ToC) is “an outcomes-based approach which applies critical thinking to the design, implementation, and evaluation of initiatives and programmes intended to support change in their context” [15]. A ToC describes, and often visually presents, how an initiative or programme, through specific intermediate outcomes summarized in ‘pathways of change’, brings about long-term outcomes. In addition, assumptions on what is required for changes to occur (including external conditions beyond programme control) and contextual factors that influence the ToC are made explicit [15, 16]. Using a ToC can help to plan, implement, monitor and evaluate complex interventions [15, 17, 18].

Complex interventions form systems of improvement amidst other complex systems, and the processes and outcomes of these interventions are being shaped by how these systems interact [19]. A ToC-based approach can assist in iterative monitoring and evaluation of complex interventions. Assessment of intervention outcomes at different levels and underlying assumptions over time can support reflexive learning and a better understanding of system interactions. A ToC-based approach can also support informed adaptation of (implementation of) the intervention to the context [19] and can help to study intervention scale-up [14, 20], which could potentially help in steering scale-up. Documentation of the application of ToCs in these processes, from planning to scaling up of health system strengthening interventions, has been limited [18]. This article seeks to address this gap.

This article describes the PERFORM2Scale ToC on the implementation and scale-up of the MSI between 2017 and 2021, and the reflections upon and adaptations of the ToC over time. It discusses how this assisted in monitoring and steering the scale-up of the MSI. The article thereby aims to contribute to understanding the benefits and challenges of using a ToC for monitoring and evaluating the scale-up of health system strengthening interventions.

## Methods

We first describe the main features of the intervention, the MSI, which was to be scaled. We then explain the framework that was used to steer the scale-up of the MSI and how the PERFORM2Scale ToC was developed, and by whom. Finally, we explain which processes were undertaken to reflect upon the ToC over a five years.

### The intervention

The core component of the MSI was a participatory action research cycle, in which DHMTs started with a situation analysis, after which they identified and prioritized health workforce and service delivery problems in their respective districts by using root cause or problem analysis (workshop 1). They then refined the problem analysis and developed a work plan to address the problems (workshop 2).^2^ The two workshops were conducted with participants from three DHMTs, included district-specific group discussions and work, and plenary feedback and reflective sessions, and were (initially) facilitated by consortium members. The work plans were then implemented over 8–12 months, within the district’s existing human and financial capacity. DHMTs reviewed the implementation of their work plan and reflected on the challenges and successes of implementing it. Consortium members conducted regular visits to the districts to support this process. As part of the MSI, broader reflection took place through inter-district meetings, during which three districts reflected upon each other’s progress. Based on these reflections, DHMTs could decide to adapt or drop activities, or start a new cycle addressing another problem [7].

### The scale-up framework

Following the ExpandNet framework [11], the MSI was the innovation to be scaled. The user organization was the Ministry of Health, and other ministries (the Ministry of Local Government and Rural Development in Malawi) or organizations (the Ghana Health Service). The national scale-up steering group, consisting of representatives of the user organization, was responsible for overseeing and steering the vertical scale-up: institutionalization or integration of the MSI in government policies, strategies, or guidelines. The resource team, which consisted of middle-level (health) officials, was responsible for horizontal scale-up: expanding the MSI over more districts. The resource team was supposed to gradually take over the facilitation role of consortium members in MSI implementation. The national scale-up steering groups and resource teams were established by PERFORM2Scale country research teams in 2018, after an initial context analysis and stakeholder mapping (2017). In each country, the MSI started in three districts in 2018, after which three new districts were added yearly, until nine districts per country in 2021. The plans for this horizontal scale-up, together with evolving plans for vertical scale-up, were jointly developed into a scale-up strategy by the country research team, resource team and national scale-up steering group in each country [21].

### The development of the theory of change

Given the complex nature of the PERFORM2Scale programme, in which the MSI – a complex intervention in itself – was to be implemented, horizontally scaled at the district level, and vertically scaled to ensure sustainability, the PERFORM2Scale consortium developed a ToC in 2017. The consortium members based in Ghana, Malawi and Uganda were, besides studying, also involved in steering the implementation and scale-up of the MSI. The ToC development process started with a participatory workshop, in which consortium members contributed and jointly discussed ideas in a large, emerging ToC visual. After this, a smaller group of consortium members conducted a literature review on factors influencing the scale-up of public health interventions [12], which together with previous evidence about the MSI [7] and the scale-up framework provided input into a first draft of the ToC (visual, narrative and assumptions). This draft was discussed by a small group of consortium members representing all partners, where initial findings of country context analyses were used to produce the first version of the PERFORM2Scale ToC. This first version was discussed and validated in a consortium meeting in 2018.

### Documentation of reflections on the theory of change

**Fig. 1:**
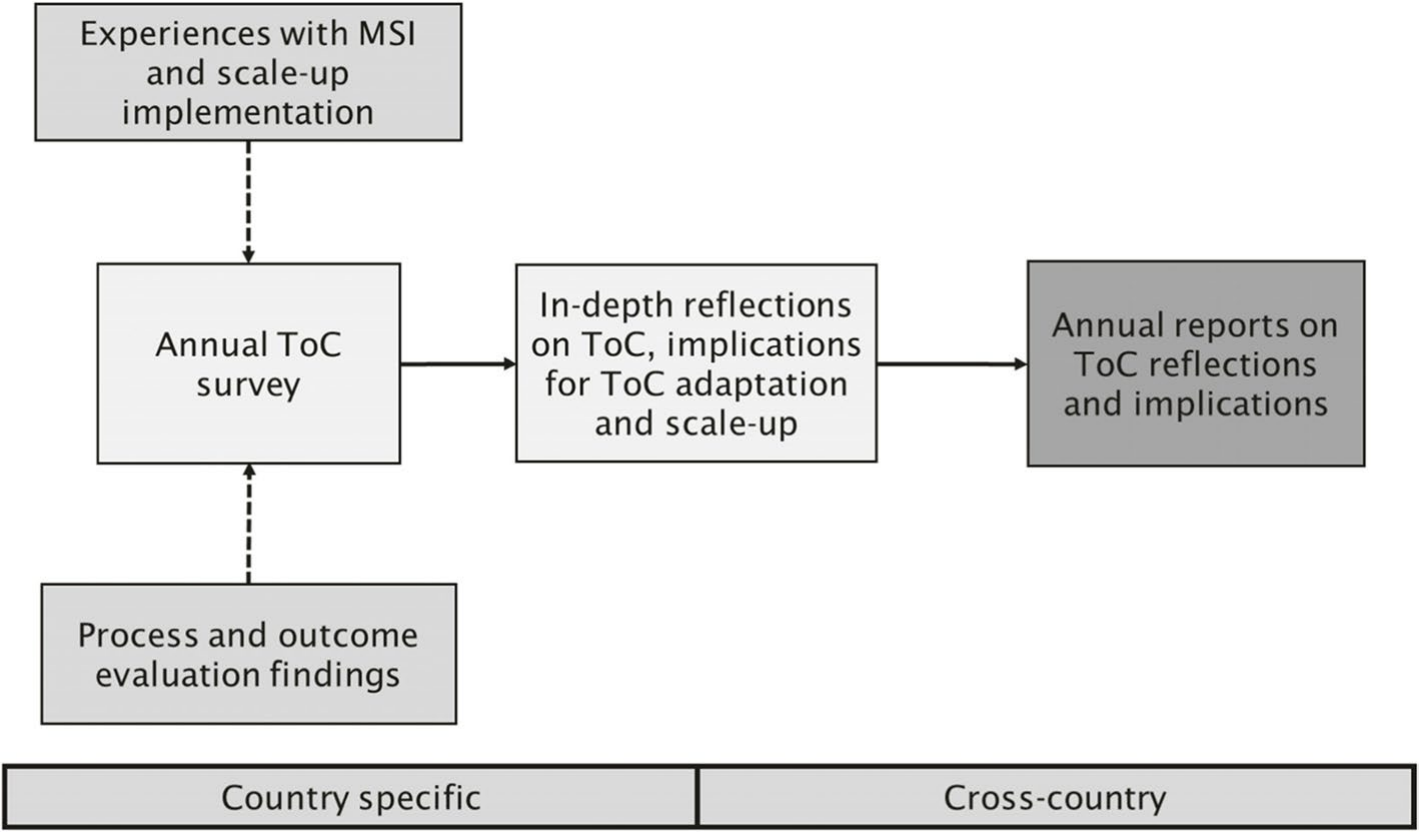
The annual ToC reflection process

From 2018 onwards, the PERFORM2Scale consortium used the ToC to monitor, evaluate and further guide the scale-up of the MSI in Ghana, Malawi, and Uganda. Reflections on the ToC and implications for MSI scale-up took place annually (2018–2021, with two reflection moments in 2020). The reflections were based on consortium members’ insights from the following processes: their involvement in MSI implementation and scale-up, and results of mixed-methods outcome and process evaluations. These evaluations, of which parts have been [4, 6, 22] and will be published elsewhere, assessed outcomes of the MSI concerning health management competencies, health workforce performance and service delivery. These evaluations also assessed how the MSI and its scale-up were implemented and why (intermediate) outcomes were or were not achieved. Keeping the (at that time) available experiences and research findings in mind, consortium members filled in an annual survey, which focused on assessing all ToC assumptions along a 5-point scale from ‘not met’ to ‘met’, about the country they worked in.^3^ The survey outcomes formed a basis for in-depth reflections on the state of MSI scale-up. This included facilitated group discussions per country, in which consortium members could explain their scoring, reflect on the ToC and identify actions to further steer the scale-up of the MSI. Key discussion points from the country groups were then fed back into plenary, after which a joint reflection on potential ToC adaptations and actions for scale-up followed. The reflections were captured in detailed annual reports (Fig. 1). The below results are based on these annual ToC reflection reports.

## Results

We first present the ToC as of March 2022 – the final month of PERFORM2Scale, specifying its adaptations over time. After this, we summarize the main findings from the consortium’s annual ToC reflections (2018–2021), including examples of actions to further steer the scale-up of the MSI and how the reflections led to adaptations of the ToC.

### The PERFORM2Scale theory of change

**Fig. 2:**
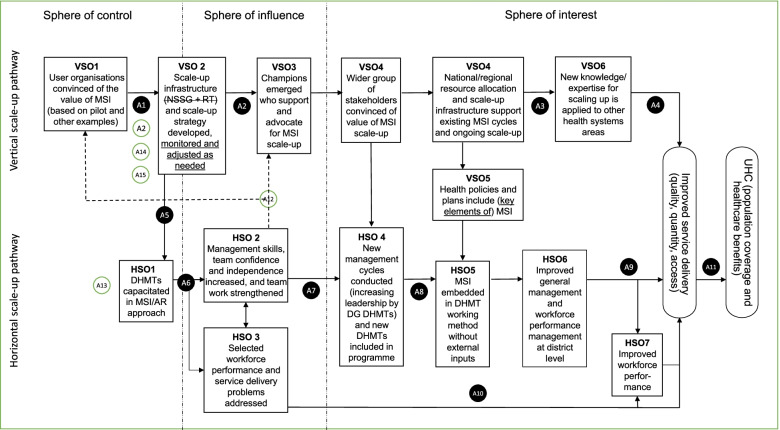
The PERFORM2Scale theory of change for scaling up management strengthening at the district level to support the achievement of universal health coverage. AR: action research; DG: district group; DHMT: district health management team; HSO: horizontal scale-up outcome; MSI: management strengthening intervention; NSSG: national scale-up steering group; RT: resource team; UHC: universal health coverage; VSO: vertical scale-up outcome. The PERFORM2Scale consortium realized that this ToC looks linear and omits some arrows; however, it was decided not to present all of them and focus on the main pathways of change. ToC adaptations are indicated in dotted lines for arrows, in underlined text and in (new) assumptions having a white instead of a black background

The PERFORM2Scale ToC (Fig. 2) consists of two pathways, referred to as the ‘horizontal scale-up pathway’ and ‘vertical scale-up pathway’. The sphere of control, influence and interest represent the different levels of expected outcomes – outcomes on which PERFORM2Scale has direct, less direct and indirect influence respectively. Assumptions are numbered and listed in Table 1. Adaptations of the ToC are indicated in both Fig. 1 and Table 1.

**Table 1:** Assumptions underlying the theory of change

Assumptions
1. Key stakeholders are convinced by the available evidence about the MSI and are *initially* (assumption 1a) and *remain* (assumption 1b) willing to collaborate with the scale-up process
2. Attention of national scale-up steering group members (assumption 2a) and resource team members (assumption 2b, added in 2018) not diverted by other priorities; low staff turnover of national scale-up steering group members (assumption 2c, added in 2020)
3. New knowledge on scale-up lessons is sufficiently well *documented* (assumption 3a) and *disseminated* (assumption 3b)
4. Sufficient opportunities to apply scale-up knowledge are available
5. DHMTs are willing to participate in the intervention even though no implementation funds are provided
6. Effective facilitation skills of the country research team (assumption 6a) and resource team (assumption 6b)* during action research cycles; work plan developed by DHMTs is feasible (time-frame, decision-authority, resources) (assumption 6c) and addresses real problems (assumption 6d)
7. DHMTs remain convinced of the value of the MSI (assumption 7a); and sufficient support is available from the resource team to support the expansion of district groups (assumption 7b)
8. Resource team members develop sufficient facilitation skills from working with new district groups (assumption 8a); low turnover of resources team members (assumption 8b)
9. DHMT remains key organisational structure at sub-national level (assumption 9a); DHMT works as a team (assumption 9b); low turnover of DHMT members (assumption 9c); decision-space does not decrease (assumption 9d)
10. DHMTs' involvement in this project, with the consequent opportunity costs, does not undermine (through possible diversion in project activities) health service delivery
11. Service delivery plans remain in line with health care needs
12. New knowledge on MSI lessons is sufficiently well *documented* (assumption 12a, added in 2019) and *disseminated* to relevant stakeholders (assumption 12b, added in 2019)
13. The MSI is a scalable intervention and, if needed, further adapted to the context in which it is implemented (added in 2021)
14. There is an understanding of power relationships between key stakeholders, which could potentially hinder or facilitate scale-up
15. Windows of opportunity to integrate (parts of) the MSI in existing structures and strategies are identified and used (added in 2021)

*DHMT* District health management team, *MSI* Management strengthening intervention

^*^ In 2019, the assumption ‘Effective facilitation skills during action research cycles’ was specified to refer to both the country research team and the resource team

The horizontal scale-up pathway started with PERFORM2Scale-facilitated capacity strengthening of DHMTs (three per country – referred to as district group 1) in the action research cycle approach. During the action research cycles, selected health workforce and service delivery problems were addressed. This would lead to improved management skills and competencies, including improved teamwork, and these outcomes would reinforce addressing workforce and service delivery problems. In year 2 of the programme, these districts started new or adjusted action research cycles, while a second district group (again, three districts per country) started the first cycle. In year 3, district groups 1 and 2 started their third and second action research cycles, respectively, and district group 3 the first. This horizontal scale-up – in which learning between districts was facilitated through inter-district meetings, together with repeated action research cycles within the districts – would make the MSI embedded in the DHMTs’ routine working without external inputs. The ongoing collective use of the MSI across an expanding number of district groups would contribute to improved general and workforce performance management at the district level, which would lead to improved workforce performance and improved service delivery. Improved service delivery would contribute to the achievement of universal health coverage.

The vertical scale-up pathway started with the outcome that the user organizations are convinced of the value of the MSI. To reach this outcome, stakeholders were engaged based on evidence from the MSI pilot (2011–2015) and similar interventions. This enabled PERFORM2Scale to establish the scale-up infrastructure (the national scale-up steering group and resource team), which was envisioned to develop country specific scale-up strategies. Assuming the experiences and outcomes from the first MSI cycles were positive, champions for scale-up emerging from within or around the national scale-up steering group and resource team would activate a wider group of stakeholders who are convinced about the value of the MSI scale-up. This stakeholder support would be essential for the national scale-up steering group and resource team to continue horizontal scale-up of the MSI and to ensure that health policies, plans, and resource allocation at national or regional levels support the ongoing scale-up of the MSI. The knowledge gained from the scale-up of the MSI would support the effective scale-up of other health system strengthening interventions, resulting in improved service delivery and contributing to universal health coverage.

The context in which the scale-up of the MSI was situated differed per country. In Ghana and Uganda, decentralization has been well-established for many years. In Malawi, a process of decentralization is ongoing [23]. However, DHMTs’ decision space is still limited in Ghana compared to the other two countries. I In Malawi, decision space has increased on paper, but in many cases not yet in practice [23]. In Ghana and Malawi, national health policies refer to strengthening of leadership and governance (Ghana) and leadership and management (Malawi) at all levels of the health system ( [24], p. 47, [25], p. 24), while in Uganda, a reference is made to strengthening district health systems in line with decentralization ( [26], p. 14).

The following two sections present summaries of the consortium’s annual ToC reflections and subsequent actions to further steer the scale-up of the MSI over time, with a focus on the spheres of control and influence. The resulting adaptations of the ToC are presented in boxes.

### Reflections on the horizontal scale-up pathway

#### DHMTs’ positive experiences with the MSI steered horizontal scale-up

In all countries, from 2018 onwards, DHMTs were trained in the MSI/ action research approach (Fig. 2, HSO1). In all countries, the annual ToC reflections revealed that DHMTs’ willingness to participate in the MSI increased over time (A5) – despite the Covid-19 pandemic delaying implementation in 2020–21. This willingness was partly because of positive experiences of (an increased number of) DHMTs in terms of problem analysis, problem-solving and teamwork (HSO2). Shared evidence on action research outcomes from their own or other districts (HSO3), e.g., improved case detection of certain diseases in Ghana, also increased DHMTs’ willingness to participate in the MSI.

**Table:** 

Adaptations of the ToC	The confirmation of the expected outcomes HSO1-3 over time, and the consequent reinforcement of assumption 5 required no change to the ToC

#### Adaptation of action cycle length was needed to embed the intervention in existing planning cycles

In 2018, when district groups 1 implemented the action research cycles for the first time, consortium members from all countries observed that the initial length of the action research cycle (8 months) was not in line with DHMTs’ annual planning cycles (12 months). To ensure that DHMTs remained convinced of the value of the MSI (A7a), the action research cycles were, as much as possible, aligned with existing planning cycles and integrated into district-level plans and budgets from 2019 (year 2 of MSI implementation).

**Table:** 

Adaptations of the ToC	In 2021, during the final annual reflection, an assumption (A13) was added at the start of the horizontal scale-up pathway to stress the importance of the MSI being a scalable intervention, and if needed further adapted to the context in which it is implemented

#### Resource teams’ capacity in MSI facilitation varied but required additional efforts from country research teams in all countries

MSI facilitation skills of the resource teams (A6b) were assessed to have improved over time in Malawi and Ghana, while this was not the case in Uganda. Consortium members working in Malawi assessed the support of the resource team to the horizontal scale-up of the MSI as increasing over time (A7b), while this was not the case in the other two countries.

In Malawi, the resource team consisted of senior staff from the central Ministry of Health and Ministry of Local Government and staff from Ministry of Health satellites (zonal offices). Most of them improved their MSI facilitation skills over time, partly because of low turnover (A8b) and championship of the steering group chair, who was the line manager of several resource team members. In Uganda, the resource team consisted of Ministry of Health officials, and while there was turnover, this was not assessed as significant over time (A8b). Facilitation of the MSI remained primarily conducted by the country research team, because the resource team was diverted by other priorities (A2b). In Ghana, the resource team mainly consisted of (a growing number of) DHMT members who were experienced in the action research cycle. They assisted the country research team in facilitating the action research cycles in other districts but, until 2020, never entirely took over. This was partly due to high turnover (A8b) and diverted priorities (A2b).

Over time, based on these reflections, country research teams in all countries made additional efforts to strengthen the resource teams, through short meetings or trainings. Resource team membership was expanded in Ghana and Malawi to have a larger pool of people to facilitate MSI workshops for DHMTs. This strategy was also tried in Uganda but did not result in more active members.

**Table:** 

Adaptations of the ToC	Assumption 2b, about the resource team not being diverted by other priorities, was added during the first ToC reflection session in 2018 In 2019, the assumption ‘Effective facilitation skills during action research cycles’ was specified to refer to both the country research team (6a) and the resource team (6b), to stress the importance of the resource team in the horizontal scale-up of the MSI Assumptions 7b and 8b, also related to the resource team, were assessed as relevant and did not change

### Reflections on the vertical scale-up pathway

#### The importance of evidence on MSI for vertical scale-up

To realize vertical scale-up of the MSI, the user organization must be convinced of the value of the MSI (Fig. 2, VSO1). In 2018, key stakeholders’ “buy-in” was assessed as low in Malawi and relatively high in Ghana and Uganda (A1a). Malawi did not participate in the pilot (2011–2015), and therefore consortium members assumed that a lack of evidence about the benefits of the MSI resulted in stakeholders being less convinced. In all countries, PERFORM2Scale made efforts to present these MSI lessons on various national platforms and via summary briefs.

**Table:** 

Adaptations of the ToC	During the annual ToC reflections in 2019, all consortium members decided to add assumption 12, indicating the importance of new knowledge on MSI lessons from district group 1, and later from the other district groups, being sufficiently well documented and disseminated to relevant stakeholders

#### Vertical scale-up seemed influenced more by priorities and mandate of people involved

Over time, however, consortium members assessed that key stakeholders’ (including user organizations’) willingness to collaborate in the scale-up process decreased in Ghana and Uganda, while this increased in Malawi. Consortium members observed that this was not particularly related to documentation and dissemination efforts of MSI lessons. In Malawi, associated with the scalability of the intervention (A13), the MSI fitted well into a prominent political priority of strengthening district-level health management in which other development partners were involved. The MSI was mainly steered by a relatively new department of the Ministry of Health that had a mandate for quality improvement. In Ghana, consortium members observed that the Ministry of Health and the Ghana Health Service were increasingly convinced about the value of the MSI over time, however, this was at the district and regional level and not at the national level. In Ghana, reflections in 2020 revealed that the national level, including the national scale-up steering group, was distanced from the MSI implementation and that the resource team (consisting of mainly DHMT members) could not influence the national level. In Uganda, key stakeholders did not see the action research approach used in MSI as significantly different from existing quality improvement interventions (again related to A13, intervention scalability), except for the focus on human resources for health and reflective practices. This hindered the vertical scale-up in the first two years.

**Table:** 

Adaptations of the ToC	The added assumption 13 on the scalability of the MSI was necessary here, in terms of relevance and relative advantage of the MSI over other management strengthening interventions

#### Power relationships became more apparent when scale-up efforts progressed

The second outcome (VSO2) of the vertical scale-up pathway initially focused on developing the scale-up infrastructure and strategy. In all countries, consortium members assessed that the national scale-up steering group had too little knowledge about the MSI and prioritized other responsibilities (including responsibilities related to Covid-19) over time (A2a), despite country research teams’ actions to engage them. This observation was strongest in Ghana. In both Malawi and Uganda, one steering group member was very active, which facilitated the scale-up of the MSI. This was related to personal leadership and to a historically good relationship between this member and one or two country research team members in both countries. From 2019 onwards, the importance of power relationships between (and among members of) country research teams, resource teams, national scale-up steering groups and other key stakeholders became more apparent. Based on this observation, the consortium spent time on further stakeholder analysis, understanding power relationships and discussing how to act upon them in each country. For example, concerning new knowledge on MSI lessons being documented and disseminated (A12), consortium members discussed whether they targeted the right stakeholders and considered what evidence is perceived as more important (e.g., qualitative versus quantitative data) and how and by whom evidence was presented.

**Table:** 

Adaptations of the ToC	Following the above, during the final ToC reflections in 2021, it was decided to add a specific assumption on the understanding of power relationships between key stakeholders, which could potentially hinder or facilitate scale-up (A14). This concerned, for example, power relationships between district and national level officials or different ministries involved in MSI scale-up In addition, it was argued that the development of the scale-up infrastructure could use existing structures with potentially more mandate or decision-influencing power, such as technical working groups, depending on the context. Therefore, a reference to the national scale-up steering group and resource team was deleted in VSO2

#### A strategy for vertical scale-up does not entirely lie in PERFORM2Scale’s sphere of control

From 2018 until 2020, the scale-up strategy mainly focused on horizontal scale-up in all countries. In Malawi and Uganda, plans for vertical scale-up started to be developed in 2020. In Malawi, the Ministry of Health plans to integrate the MSI into the satellite structure that provides technical assistance to DHMTs. The department of the Ministry of Health steering the MSI had a particular interest in reviving this satellite structure in light of quality improvement, and the MSI fitted well in this. In Uganda, only specific elements of the MSI are integrated into the new quality improvement strategic plan and framework of the Ministry of Health, published in 2021 [27]. These plans for vertical scale-up were developed over various meetings with key stakeholders from the Ministry of Health and the Ministry of Local Government, and they were related to windows of opportunities appearing at certain times.

**Table:** 

Adaptations of the ToC	In 2020, the consortium agreed that developing a scale-up strategy (VSO2) is not solely within the ToC sphere of control, which it was initially expected to be. It also concluded that additions were needed in the formulation of this outcome. At first, the formulation of this outcome only stressed the development of the scale-up infrastructure and strategy; in 2020 monitoring and adjusting them were added In 2021, the consortium decided to add one more explicit assumption in the ToC (A15), about windows of opportunity to integrate (parts of) the MSI in existing structures and strategies being identified and used

#### More champions are needed to realize vertical scale-up

Regarding champions (VSO3), all countries observed emerging champions at the district level (DHMT members) and at the regional level (in case of Ghana), but no champions (Ghana) and very few champions (Malawi and Uganda) emerged at the national level. As indicated above, in Malawi and Uganda, the NSSG chairs were championing the scale-up of the MSI. In Malawi, after a change in the political landscape because of elections in 2020, this champion disappeared. The strong resource team was able to maintain attention to the scale-up of the MSI within the Ministry of Health and the Ministry of Local Government. According to consortium members’ assessments over time, the scattered development partner landscape, characterized by little coordination and interest in each other’s projects, resulted in having no champions at the national level outside the national scale-up steering group and resource team in Malawi. It, therefore, remains to be seen whether vertical scale-up will be realized. Consortium members realized that there was a continuous need to inform and map the interests of potential champions.

**Table:** 

Adaptations of the ToC	Concerning the emergence of champions, assumption 2 remained relevant over time, as well as the added assumption on power relationships (A14)

## Discussion

At the start of PERFORM2Scale, the development of the ToC assisted in creating a common vision for the programme. We triangulated consortium members’ experience-based knowledge^4^ with research evidence during ToC development [15, 28]. Following Stein and Valter’s broad categories of the purpose of ToCs, the PERFORM2Scale ToC articulated expected processes and outcomes that were reviewed over time (monitoring and evaluation purpose) and served as a thinking tool, helping the consortium to clarify and develop the theory behind the programme (learning purpose) [29].

The annual ToC reflections proved valuable in gaining a nuanced understanding of why and how change does (and does not) happen, allowing to respond to (unpredictable) developments [29, 30], for example, through adaptations of the MSI or approaching stakeholders who were not approached before, to steer the scale-up of the MSI in Ghana, Malawi and Uganda. This iterative process was particularly based on learnings about interlinkages between horizontal and vertical scale-up, the (behaviour of) key stakeholders and champions involved and the health system and overall context in which the scale-up of the MSI took place. As the scale-up of the MSI was a system of improvement amidst other complex systems [19], expectedly, the consortium could not address all factors that hindered scale-up.

While the initial PERFORM2Scale ToC, following the scale-up framework adapted from ExpandNet, focused on key stakeholders involved in the implementation and scale-up of the MSI, it less explicitly focused on the relationships between those stakeholders. Over the course of PERFORM2Scale, scale-up was influenced by power relationships between different stakeholders, maybe more than initially thought. Scholars recognize the overwhelming – or even impossible – task of selecting the critical assumptions in a ToC [31]. Three explicit assumptions, including one about power relationships, were added towards the end of PERFORM2Scale. Practitioners’ experiences with using ToCs have revealed that certain assumptions ‘simply can’t be written down’ because of their sensitivity [15, 30]. In our case, the set of initial assumptions might have been a result of the composition of the consortium, with a stronger emphasis on expertise in human resource management and management strengthening than in political economy analysis.

We also recognize that the PERFORM2Scale ToC is a simplified representation of a complex scale-up process. Nevertheless, the annual ToC reflections went far beyond this simplified representation and enabled studying the scale-up of the MSI [14, 20]. We believe that a ToC-based approach, in which outcomes and assumptions are continuously reflected upon, could potentially increase chances of successful scale-up. At the time of writing, however, it remains to be seen whether the MSI will be institutionalized or integrated into government policies, strategies or guidelines and resource allocation in any of the countries involved in PERFORM2Scale.

It is clear from the literature that it is advisable to develop a ToC and conduct subsequent reflections with a wide variety of stakeholders to extend ownership of the intervention, its implementation and evaluation [15, 17, 32]. While our consortium consisted of researchers who also had a role in intervention implementation and scale-up, key stakeholders (user organizations) and end-users (DHMTs) in each of the three countries were not involved in the ToC process, and no country-specific ToCs were developed. Reflections on the scale-up of the MSI and subsequent actions would probably have been different – more context-specific and maybe more successful – if these people had been involved. In our case, we can speak about ‘evaluator ownership’ of the ToC [32], mainly because of insufficient time and financial resources to include others in this process. Key stakeholders and end-users in the three countries were only implicitly involved in reflecting on parts of the theory of change when participating in the initial context analysis (which informed ToC development) and outcome and process evaluation interviews or group discussions (which informed annual ToC reflections within the consortium).

A limitation of this article is that it does not describe the methods that were used for the outcome and process evaluation of the scale-up of the MSI in Ghana, Malawi and Uganda. They included a variety of quantitative and qualitative methods that were used over time and that focused on the MSI, its scale-up, and the systems in which this took place; as suggested for monitoring and evaluating the scale-up of complex interventions [33–36]. We did describe how implementation experiences and findings from the research were used to reflect upon the hypothesised pathways of change, to explain observed changes over the years, and to further steer the scale-up of the MSI. The PERFORM2Scale ToC was therefore used as a resource for structuring and guiding monitoring and evaluation and will also be used in the programme’s final evaluation [33]. Through ToC reflections over time, monitoring and evaluation could become a purposeful part of the scale-up process [35]. It facilitated the consortium’s learning about the process of implementation and scale-up, about being flexible (the importance of adaptation) and being in tune with contextual changes and stakeholders involved [18, 36].

Learnings about the annual ToC reflections were that they were enriched by using multiple participatory methods, including individual scoring exercises, country and consortium-wide group discussions and visualizations; guided by a ‘reflection facilitator’ [15] part of PERFORM2Scale, but not directly involved in steering the scale-up of the MSI in one of the countries. It required continued commitment to reflection and learning from all consortium members, for which resources need to be available [15, 30]. Rather than seeing the ToC as a product, it has been a process, a way of working, in the consortium’s aim to study and steer the scale-up of the MSI.

## Conclusions

The PERFORM2Scale’s experience has shown the benefits and challenges of using a ToC for monitoring and evaluating the implementation and scale-up of a health management strengthening intervention in Ghana, Malawi and Uganda. A ToC-based approach in implementation research and practice can enhance continuous reflection on how change happens (or not), allowing adaptations in the scale-up process to steer horizontal and vertical scale-up. It is particularly important to monitor critical assumptions of a ToC, taking into account the political economy. ToC-based approaches should include a variety of stakeholders, including policy makers, implementers and researchers, and require their continued commitment to reflection and learning and is thus resource intense. We conclude that ToC-based approaches are valuable for iterative monitoring and evaluation of the implementation and scale-up of health system strengthening interventions, and can potentially increase chances for successful scale-up.

## Data Availability

The datasets generated and analysed during the current study are available from the corresponding author on reasonable request.

## Abbreviations

DHMT: District health management teams
MSI: Management strengthening intervention
ToC: Theory of change

## Footnotes

1 PERFORM2Scale consortium partners are: Liverpool School of Tropical Medicine (lead), KIT Royal Tropical Institute, Makerere University – School of Public Health, Maynooth University, Reach Trust, Swiss Tropical and Public Health Institute, Trinity College – Centre for Global Health, University of Ghana – School of Public Health.

2 More information about the focus of work plans in the three countries can be found in Country reports 2021.

3 European partners were paired with country research teams in the three different countries throughout PERFORM2Scale, except KIT Royal Tropical Institute, of which colleagues did not participate in the scoring and reflection exercise, but facilitated the exercise.

4 In relation to health system strengthening interventions in the respective country contexts.
